# Pro-opiomelanocortin and its Processing Enzymes Associate with Plaque Stability in Human Atherosclerosis – Tampere Vascular Study

**DOI:** 10.1038/s41598-018-33523-7

**Published:** 2018-10-10

**Authors:** Petteri Rinne, Leo-Pekka Lyytikäinen, Emma Raitoharju, James J. Kadiri, Ivana Kholova, Mika Kähönen, Terho Lehtimäki, Niku Oksala

**Affiliations:** 1 Research Center for Integrative Physiology and Pharmacology, Institute of Biomedicine, University of Turku, Turku, Finland; 2 Turku Center for Disease Modeling, University of Turku, Turku, Finland; 3 Department of Clinical Chemistry, Fimlab Laboratories and Finnish Cardiovascular Research Center-Tampere, Faculty of Medicine and Life Sciences, University of Tampere, Tampere, Finland; 4 Department of Pathology, Fimlab Laboratories and Finnish Cardiovascular Research Center-Tampere, Faculty of Medicine and Life Sciences, University of Tampere, Tampere, Finland; 5 Department of Clinical Physiology, University of Tampere and Tampere University Hospital, Tampere, Finland; 6 Department of Surgery, Tampere University Hospital, Tampere, Finland and Faculty of Medicine and Life Sciences, University of Tampere, Tampere, Finland and Finnish Cardiovascular Research Center-Tampere, Tampere, Finland

## Abstract

α-melanocyte-stimulating hormone (α-MSH) is processed from pro-opiomelanocortin (POMC) and mediates anti-inflammatory actions in leukocytes. α-MSH also promotes macrophage reverse cholesterol transport by inducing ATP-binding cassette transporters ABCA1 and ABCG1. Here we investigated the regulation of POMC and α-MSH expression in atherosclerosis. First, transcript levels of POMC and its processing enzymes were analyzed in human arterial plaques (n = 68) and non-atherosclerotic controls (n = 24) as well as in whole blood samples from coronary artery disease patients (n = 55) and controls (n = 45) by microarray. POMC expression was increased in femoral plaques compared to control samples as well as in unstable advanced plaques. α-MSH-producing enzyme, carboxypeptidase E, was down-regulated, whereas prolylcarboxypeptidase, an enzyme inactivating α-MSH, was up-regulated in unstable plaques compared to stable plaques, suggesting a possible reduction in intraplaque α-MSH levels. Second, immunohistochemical analyses revealed the presence of α-MSH in atherosclerotic plaques and its localization in macrophages and other cell types. Lastly, supporting the role of α-MSH in reverse cholesterol transport, POMC expression correlated with ABCA1 and ABCG1 in human plaque and whole blood samples. In conclusion, α-MSH is expressed in atherosclerotic plaques and its processing enzymes associate with plaque stability, suggesting that measures to enhance the local bioavailability of α-MSH might protect against atherosclerosis.

## Introduction

Atherosclerosis is a chronic inflammatory disease that is driven by persistent lipid overload, immune activation and accumulation of monocyte-derived macrophages in the growing lesions^1,2^. The disease progression is largely a matter of unresolved inflammation that is characterized by continuous recruitment of pro-inflammatory leukocytes, macrophage proliferation and defective efferocytosis to clear apoptotic cells. These events gradually lead to expansion of necrotic core and thinning of fibrous cap overlying the core, which are hallmarks of a vulnerable plaque at high risk to rupture and cause acute complications. Therefore, investigating the molecular and cellular processes that contribute to the disease progression is fundamental and aids to identify novel therapeutic targets that could boost inflammation resolution and provide plaque-stabilizing effects.

Pro-opiomelanocortin (POMC) is a multipotent prohormone that gives rise to smaller peptide hormones through post-translational processing^3^. These peptides include adrenocorticotropic hormone (ACTH) and α-, β- and γ-melanocyte-stimulating hormones (MSH) that are collectively called melanocortins. POMC is predominantly expressed in the pituitary gland, particularly in the corticotrophs and melanotrophs of the anterior and intermediate lobes. It is also found in distinct nuclei in the central nervous system, in a variety of peripheral tissues (*e.g*. skin, kidney, and liver) and in the cells of the immune system including monocytes and macrophages^4^. According to the current understanding, POMC-derived peptides are released into circulation by the pituitary gland, whereas in other tissues, POMC is processed to act in an autocrine or paracrine manner. The formation of biologically active melanocortins requires well-coordinated actions by several enzymes^3^. The expression of these enzymes is spatially and temporally regulated and co-localizes in POMC-expressing cells. The post-translational processing is initiated by prohormone convertases, PC1/3 and PC2, the former generating larger fragments from the POMC and the latter continuing the cleavage into smaller peptides such as ACTH_1–17_. Further processing of ACTH_1–17_ to yield mature α-MSH (Ac-ACTH_1–13_-NH2) is driven by carboxypeptidase E (CPE) and α-amidating monooxygenase (PAM). α-MSH has a very short half-life since it is rapidly metabolized by an enzyme, prolylcarboxypeptidase (PRCP), which inactivates α-MSH by removing the C-terminal valine residue^5^.

POMC-derived melanocortins bind and activate a family of G-protein coupled melanocortin receptors (MC1R-MC5R) to exert their physiological functions. Since the early discoveries of the pigmentary function of α-MSH and the role of ACTH in glucocorticoid production, our understanding of the melanocortins and their receptors has greatly advanced and we now appreciate that they contribute to diverse physiological functions ranging from appetite control to blood pressure regulation and immunomodulation^6^. For example, the role of α-MSH extends well beyond skin pigmentation. Through the interaction with its natural receptor MC1R, α-MSH not only elicits potent and well-established anti-inflammatory responses in leukocytes but also regulates endothelial NO production and vascular tone^7–9^. We also recently identified that α-MSH promotes macrophage cholesterol efflux and thereby protects against foam cell formation by activating MC1R^10^. Mechanistically, α-MSH was shown to induce the expression of ATP-binding cassette transporters ABCA1 and ABCG1, which mediate macrophage cholesterol efflux onto high-density lipoprotein (HDL) particles^11^. This is an important mechanism that helps to resolve cholesterol accumulation and inflammation in atherosclerotic plaques, thus promoting plaque regression, and could ideally complement other lipid-lowering therapies in the management of atherosclerotic cardiovascular disease^12,13^.

Owing to its role in the control of inflammation, vascular tone and inflammation, α-MSH possesses numerous properties that could favorably modulate the progression of atherosclerosis and stabilize existing plaques. This prompted us to investigate whether POMC, α-MSH and the related processing enzymes are expressed locally in human atherosclerotic plaques. Based on our previous findings showing that genetic overexpression of α-MSH and pharmacological administration of its stable analogue protect against atherosclerosis^14,15^, we hypothesized that the expression of POMC-derived α-MSH associate with plaque stability and may become disturbed during progression to unstable advanced lesions.

## Results

### POMC expression is up-regulated in unstable atherosclerotic plaques

**Figure 1:**
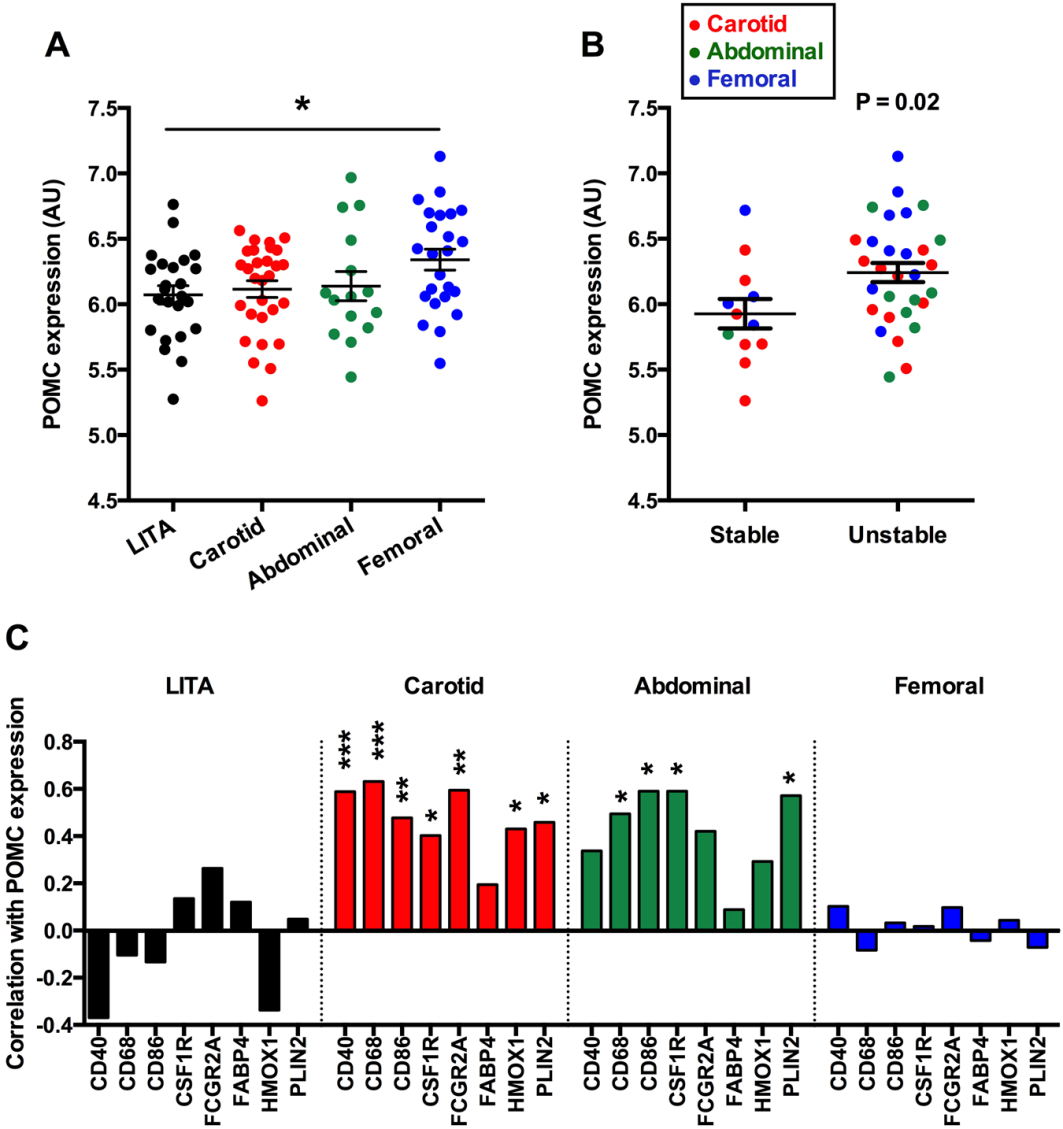
Expression of pro-opiomelanocortin (POMC) in human atherosclerotic plaques. (**A**) Pro-opiomelanocortin (POMC) expression in atherosclerosis-free control arteries (left internal thoracic artery; LITA, n = 24) and in endarterectomy samples from the carotid artery (n = 29), abdominal aorta (n = 15) and femoral artery (n = 24). LITA samples served as controls and they were histologically verified to be atherosclerosis-free. *P < 0.05 for the indicated comparison (one-way ANOVA and Bonferroni *post hoc* test). (**B**) POMC expression in stable and unstable plaque phenotypes in a subgroup of advanced plaques (stage V and VI). Exact P-value (two-tailed Student’s t test) is given in the graph. (**C**) Correlation between POMC mRNA levels and general macrophage markers in control LITA samples and in carotid, abdominal and femoral plaque samples. Pearson correlation coefficient (r) values are presented in the column graphs. *P < 0.05, **P < 0.01 and ***P < 0.001 for correlation significances.

We first aimed to test the hypothesis that POMC and its processing product α-MSH are expressed in human atherosclerotic plaques. In a microarray-based analysis, which has been previously validated and shown good accuracy in comparison with real-time quantitative PCR^16^, POMC was expressed in all plaque samples including carotid artery, abdominal aortic and femoral artery plaques. The expression level of POMC in carotid or abdominal samples was not significantly different in comparison with atherosclerosis-free left internal thoracic artery (LITA) samples (Fig. 1A). However, femoral arterial plaques showed subtle but significant up-regulation (fold change, FC = 1.2, P = 0.04) of POMC compared to control samples (Fig. 1A). Interestingly, when comparing stable and unstable advanced lesions, POMC transcript levels were increased in unstable plaque samples (Fig. 1B).

**Figure 2:**
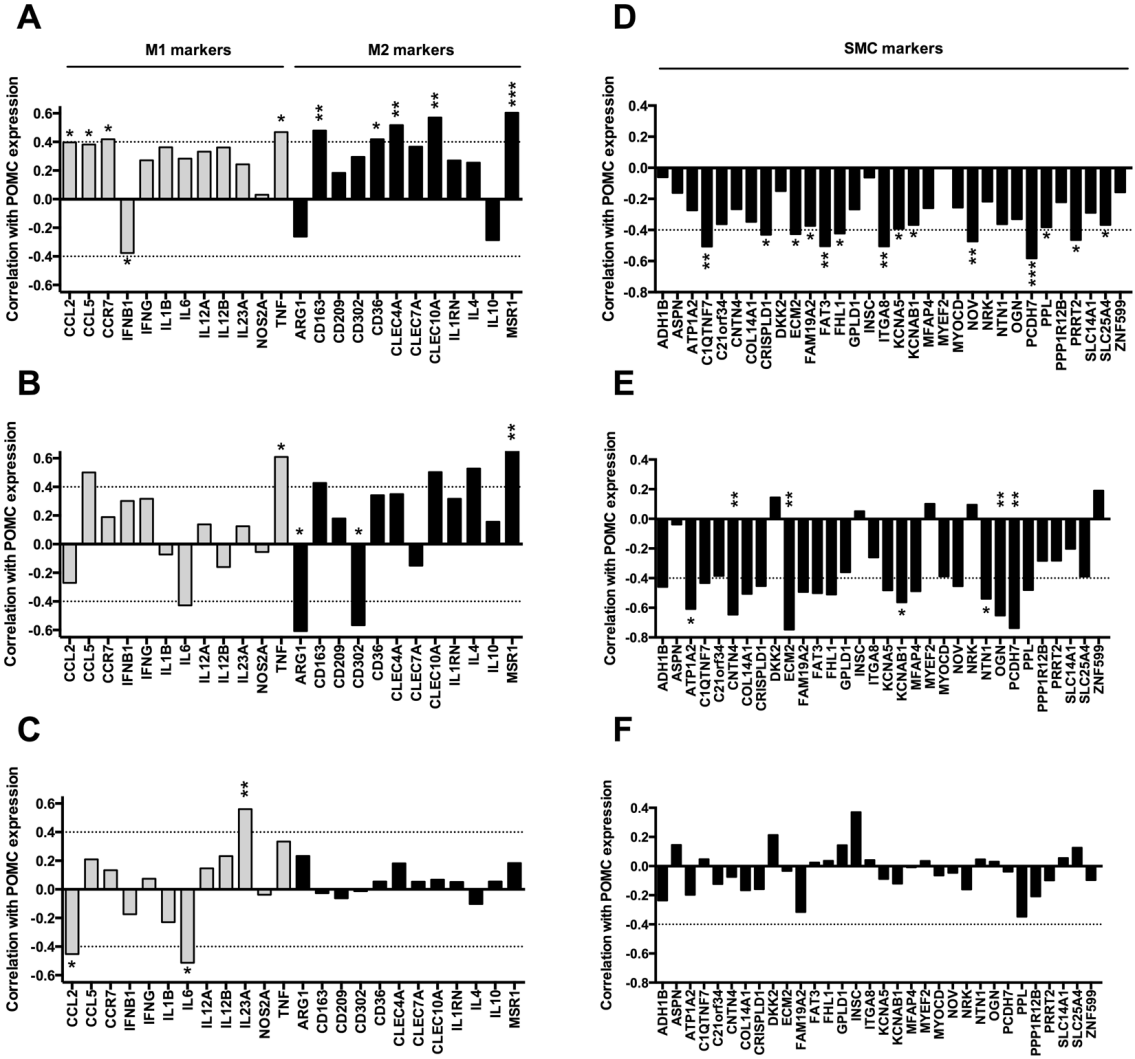
POMC expression in human atherosclerotic plaques is positively correlated with M1 and M2 macrophage markers and negatively with smooth muscle cell markers. (**A–C**) Correlation between POMC mRNA levels and established M1 (grey columns) and M2 (black columns) macrophage markers in carotid (**A**), abdominal (**B**) and femoral (**C**) plaque samples. (**D–F**) Correlation between POMC mRNA levels and smooth muscle cell markers in carotid (**A**), abdominal (**B**) and femoral (**C**) plaque samples. Pearson correlation coefficient (r) values are presented in the column graphs. *P < 0.05, **P < 0.01 and ***P < 0.001 for correlation significances.

**Figure 3:**
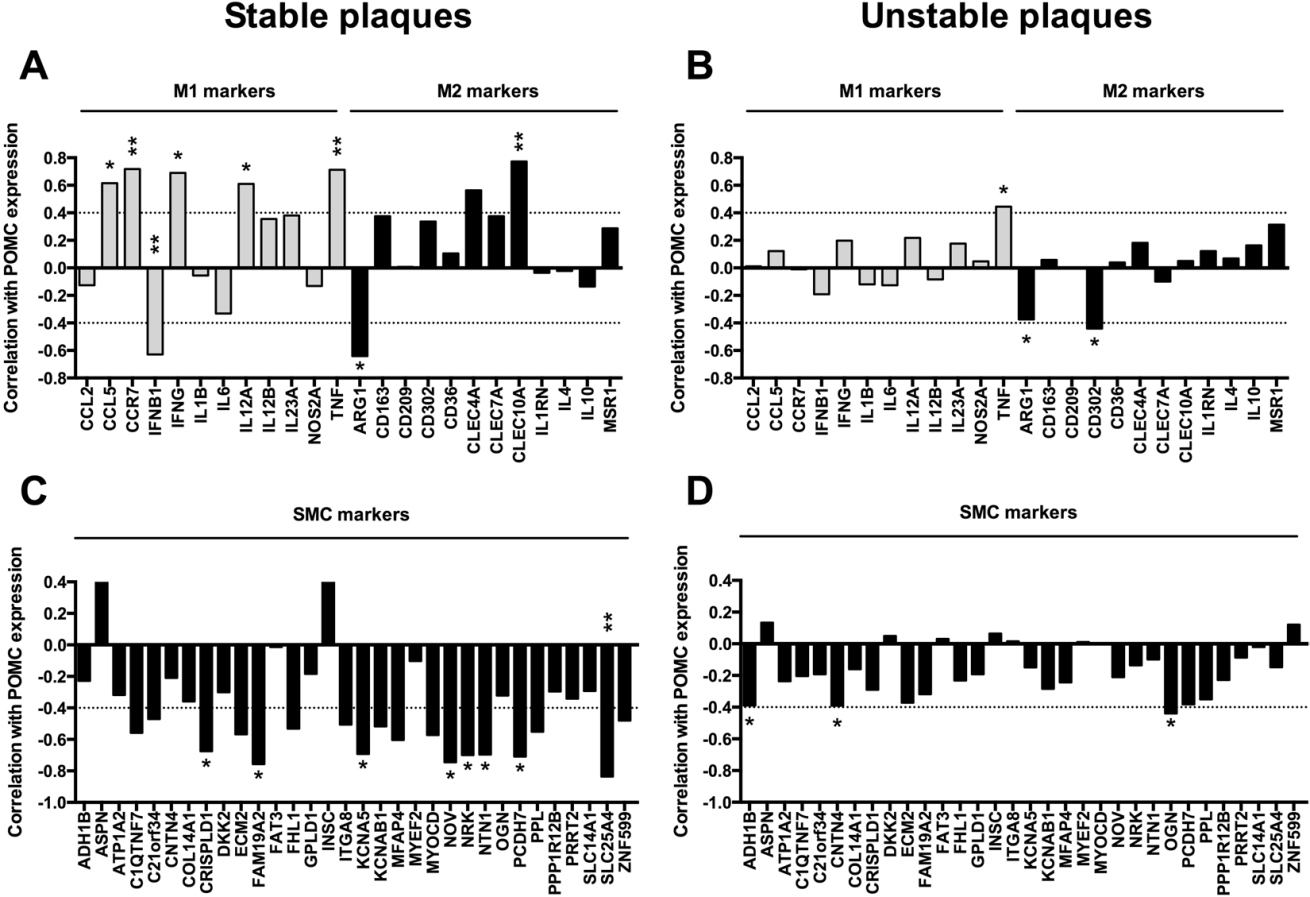
Association of POMC transcript with M1/M2 macrophage and smooth muscle cell markers is attenuated in unstable advanced plaques. (**A,B**) Correlation between POMC mRNA levels and established M1 (grey columns) and M2 (black columns) macrophage markers in stable and unstable plaques. (**C,D**) Correlation between POMC mRNA levels and smooth muscle cell markers in stable and unstable advanced plaques. Pearson correlation coefficient (r) values are presented in the column graphs. *P < 0.05 and **P < 0.01 for correlation significances.

Gene association analyses revealed that POMC expression correlated positively with general macrophage markers such as CD68 and CSF1R in carotid and abdominal plaque samples (Fig. 1C), while the associations were absent in femoral plaques and in LITA samples (Fig. 1C). POMC also positively correlated with established signature markers of pro-inflammatory M1 and anti-inflammatory M2 type macrophages^17,18^ particularly in carotid plaques (Fig. 2A–C), but the association patterns were not markedly different between M1 and M2 markers. In contrast, the associations between POMC and SMC markers was negative in carotid and abdominal plaque samples (Fig. 2D,E), while femoral plaques showed relatively weak correlation with SMC markers (Fig. 2F). In line with these results, similar analyses in stable and unstable samples revealed that the strength of correlation with M1/M2 macrophage and SMC plaque signatures was weaker in unstable advanced plaques (Fig. 3). Collectively, POMC was up-regulated in femoral plaques as well as in unstable plaques and these changes were associated with blunted correlation patterns with macrophage and SMC markers.

### Human and mouse atherosclerotic plaques express α-MSH

**Figure 4:**
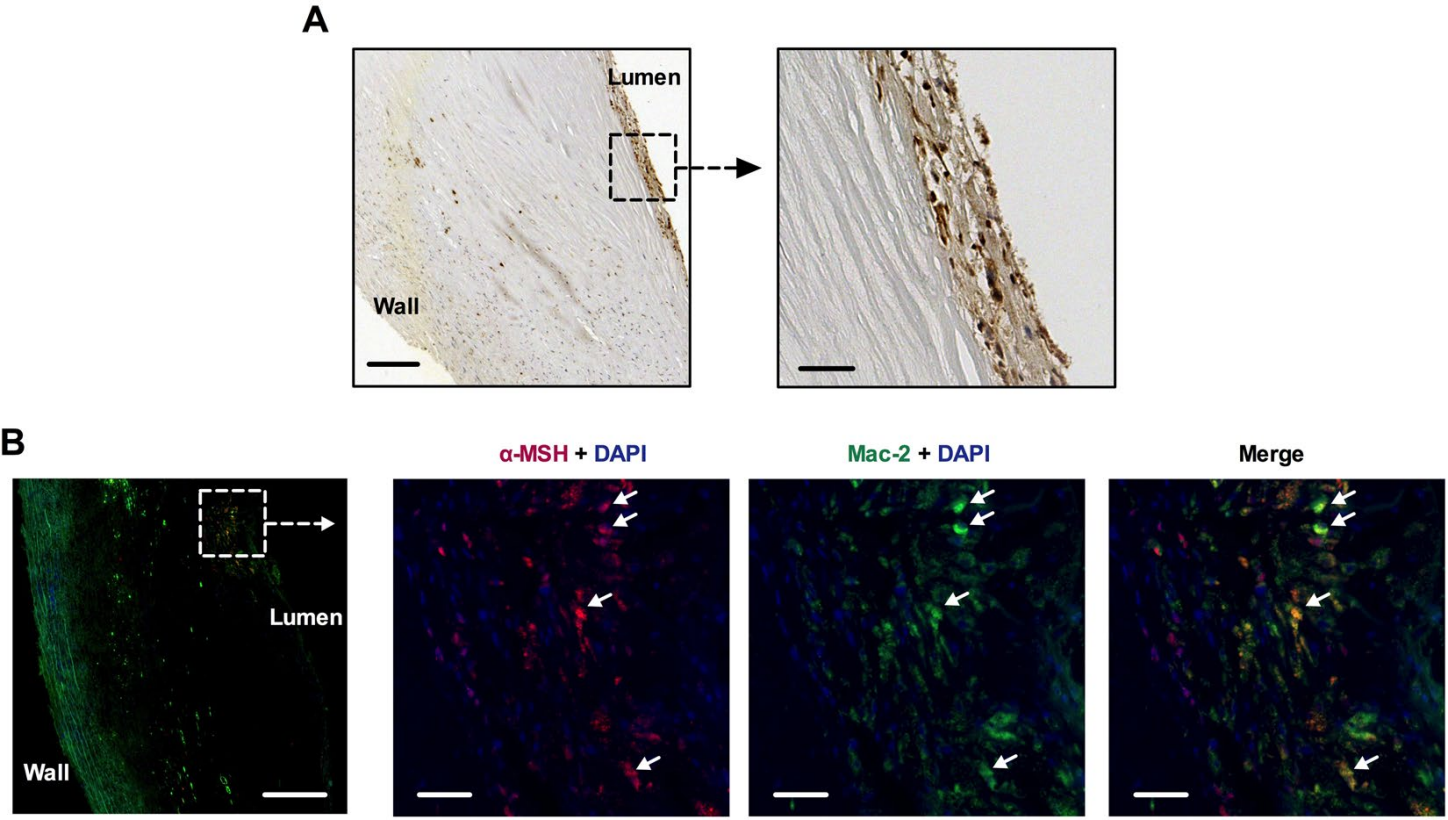
α-melanocyte stimulating hormone (α-MSH) is expressed by plaque macrophages in human atheroma. (**A**) α-MSH immunostaining (brown color) of carotid endarterectomy sample. Scale bars, 200 µm (left) and 50 µm (right). (**B**) A consecutive section of the carotid sample was immunofluorescently stained for α-MSH (red) and Mac-2 (green), and counterstained with DAPI. Cells that clearly express both α-MSH and Mac-2 are indicated by white arrows. Scale bars, 200 µm (left) and 50 µm (right).

To study whether plaque POMC expression leads to detectable traits of its main processing product α-MSH, carotid plaque sample was immunostained with an antibody against α-MSH. Conventional immunohistochemistry confirmed the presence of α-MSH in in the atherosclerotic plaque, particularly in close proximity to the arterial lumen (Fig. 4A). Double immunofluorescence further revealed that α-MSH merged with Mac-2 staining in the lesion (Fig. 4B), indicating co-localization in macrophages. Consistently, aortic root plaques from Apoe^−/−^ mice showed distinct α-MSH expression on Mac-2-positive macrophages as well as on the intimal layer (Supplementary Fig. IA), supporting our earlier finding of endothelial α-MSH expression in healthy arteries^9^. It was also found that plaque α-MSH expression reduced in advanced atherosclerosis when Apoe^−/−^ mice fed an atherogenic high-fat diet (Supplementary Fig. IB and C). In contrast to declining α-MSH amount, POMC expression was increased in the aorta of Apoe^−/−^ mice after high-fat diet (Supplementary Fig. ID).

### POMC expression correlates with reverse cholesterol transporters in atherosclerotic plaques and in whole blood samples

**Figure 5:**
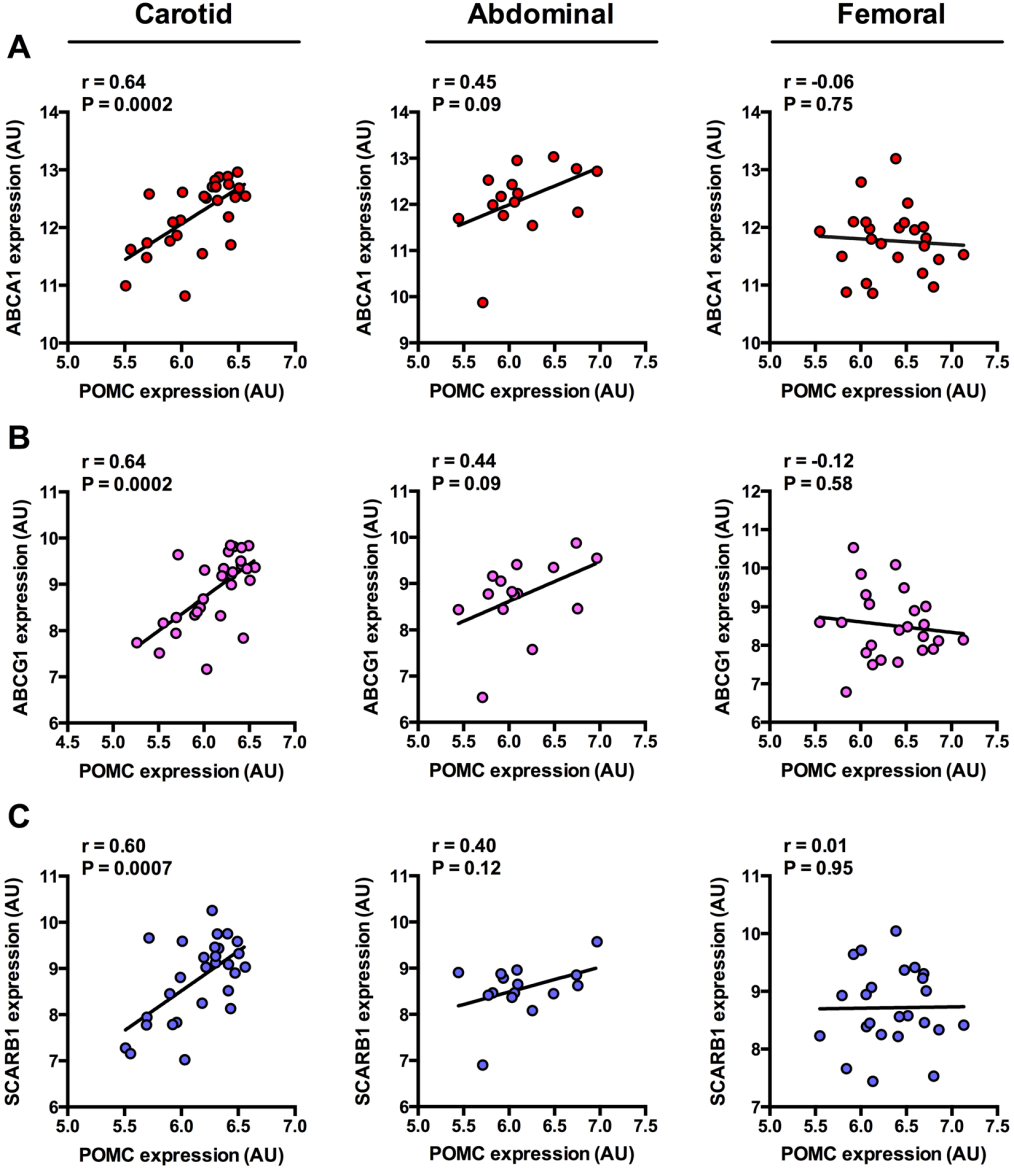
POMC expression is directly associated with reverse cholesterol transporters in carotid artery plaques. (**A**–**C**) Correlations between POMC expression and ATP-binding cassette transporter A1 (ABCA1), G1 (ABCG1) and scavenger receptor class B member 1 (SCARB1) expression in carotid (left panel), abdominal (middle panel) and femoral (right panel) atherosclerotic plaques. Pearson correlation coefficients (r) and P values are presented in the graphs.

Further screening of carotid plaque samples uncovered that POMC expression is directly associated with the reverse cholesterol transporters ABCA1, ABCG1 and scavenger receptor class B member 1 (SCARB1) (Fig. 5), which is in good agreement with our previous study demonstrating similar associations with MC1R expression^10^. Of note, the correlations with ABCA1 and ABCG1 expression were stronger (r = 0.64, P = 0.0002 for both in carotid samples) than with any of the other analyzed macrophage and SMC markers, suggesting that POMC is centrally involved in the regulation of reverse cholesterol transport. These correlation patterns were somewhat evident also in abdominal plaque samples (Fig. 5), while femoral plaques showed no clear correlation with ABCA1, ABCG1 or SCARB1 (Fig. 5).

**Figure 6:**
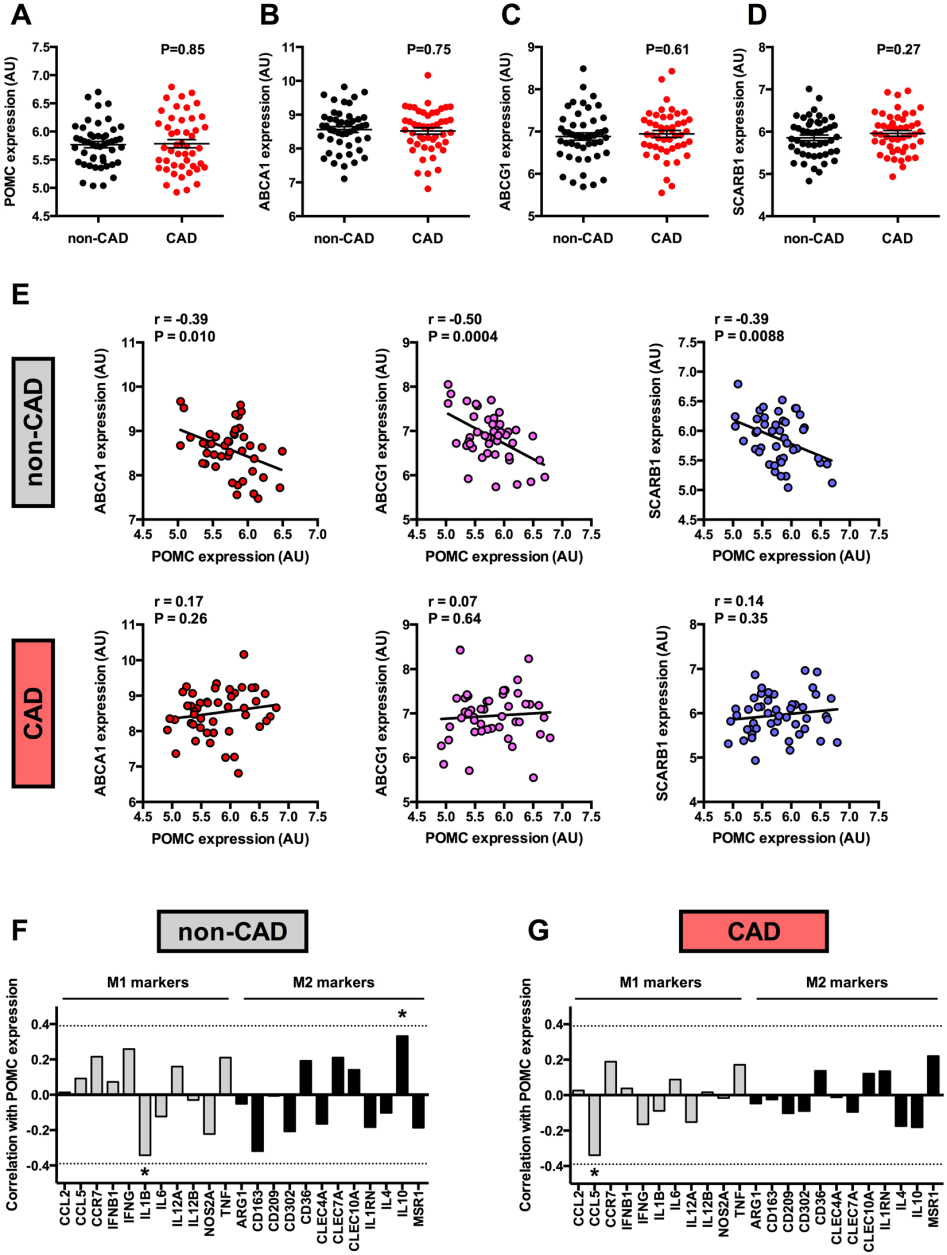
POMC expression negatively correlates with reverse cholesterol transporters in the whole blood, but the association is abolished in coronary artery disease. (**A–D**) POMC, ABCA1, ABCG1 and SCARB1 mRNA levels in whole blood samples of patients with history of coronary artery disease (CAD) and of individuals without coronary lesions (non-CAD). Exact P values (two-tailed Student’s t test) are presented in the graphs. (**E**) Correlations between POMC expression and ABCA1, ABCG1 and SCARB1 expression in whole blood samples from non-CAD and CAD individuals. Pearson correlation coefficients (r) and P values are presented in the graphs. (**F,G**) Correlation between POMC mRNA levels and established M1 (grey columns) and M2 (black columns) macrophage markers in non-CAD (n = 48) and CAD (n = 47) whole blood samples. Spearman correlation coefficient (r) values are presented in the column graphs. The dashed lines are placed at 0.39 and −0.39 that represent the correlation level between POMC and ABCA1/SCARB1. *P < 0.05 for correlation significances.

To further investigate the association between POMC and reverse cholesterol transporters, we analyzed whole blood and circulating monocyte samples from patients with coronary artery disease (CAD) and compared them with angiographically normal controls (non-CAD). The expression of POMC, ABCA1, ABCG1 or SCARB1 did not differ between CAD and non-CAD samples in either whole blood or monocyte samples (Fig. 6A–D and Supplementary Fig. IIA–D). However, POMC expression was found to negatively associate with ABCA1 (P = 0.010), ABCG1 (P = 0.0004) and SCARB1 (P = 0.0088) in the whole blood samples of non-CAD controls, while these correlations were completely absent in patients with CAD (P > 0.2 for all, Fig. 6E). The correlations with ABCA1, ABCG1 and SCARB1 were stronger compared to classical pro-inflammatory or anti-inflammatory markers (Fig. 6F,G). In non-CAD samples, POMC expression correlated negatively with pro-inflammatory IL-1β (r = −0.34, P = 0.017) and positively with anti-inflammatory IL-10 (r = 0.33, P = 0.021). In contrast to the whole blood analysis, monocyte fractions from the same individuals did not show any association between POMC expression and reverse cholesterol transporters in either non-CAD or CAD study population (Supplementary Fig. IIE). Likewise, no significant associations were noted between POMC and pro- or anti-inflammatory cytokines (Supplementary Fig. IIF and G).

### Enzymes involved in the processing of POMC are associated with atherosclerotic plaque stability

**Figure 7:**
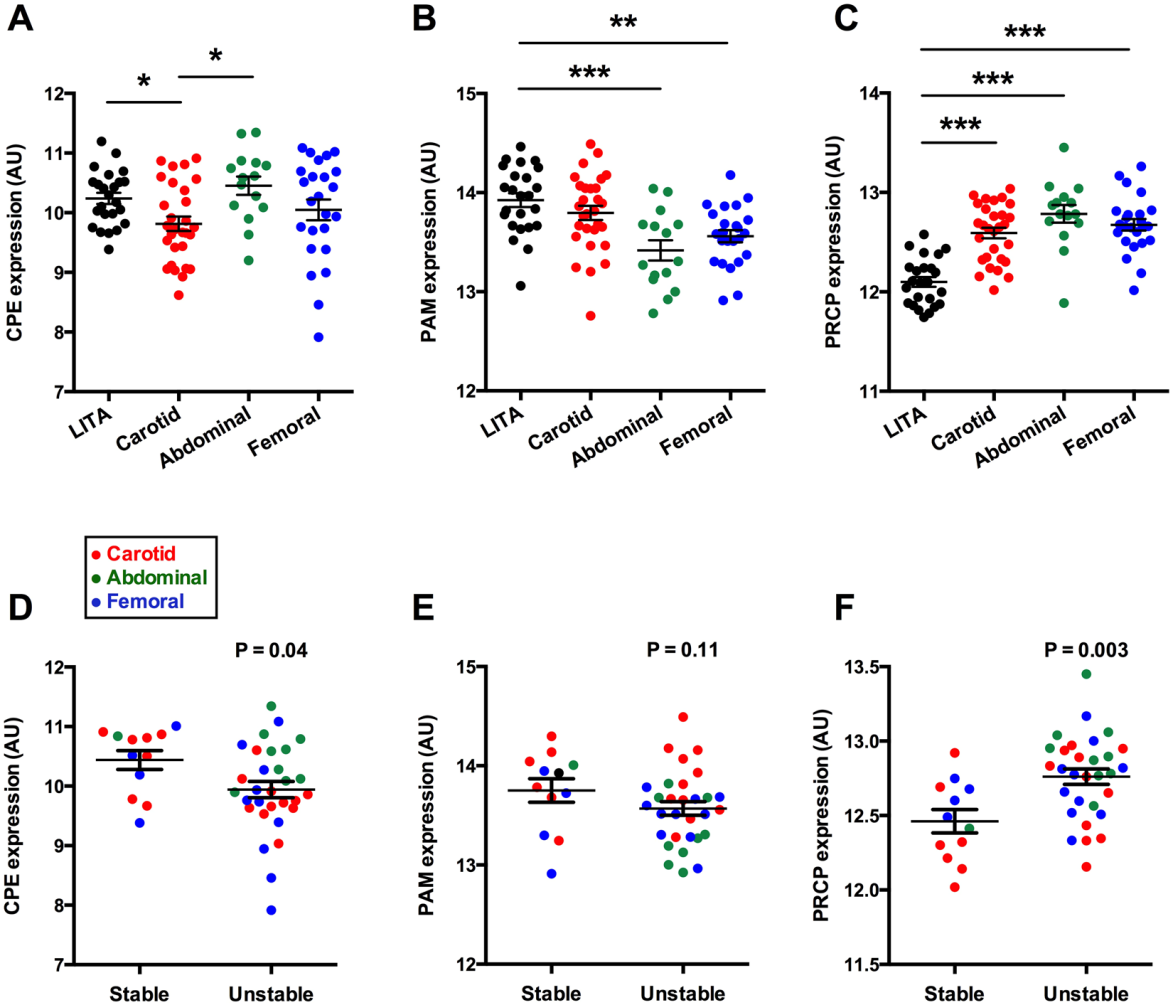
Prolylcarboxypeptidase, an enzyme degrading α-MSH, is upregulated in unstable atherosclerotic plaques. (**A–C**) The expression of CPE, PAM and PRCP expression in control arteries (LITA) and in carotid artery, abdominal and femoral atherosclerotic samples. CPE, carboxypeptidase E; PAM, peptidylglycine α-amidating monooxygenase; PRCP, prolylcarboxypeptidase *P < 0.05, **P < 0.01 and ***P < 0.001 for the indicated comparisons (one-way ANOVA and Bonferroni *post hoc* test). (**D–F**) CPE and PRCP mRNA expression in stable and unstable advanced atherosclerotic plaques. Exact P values (two-tailed Student’s t test) are given in the graphs.

**Figure 8:**
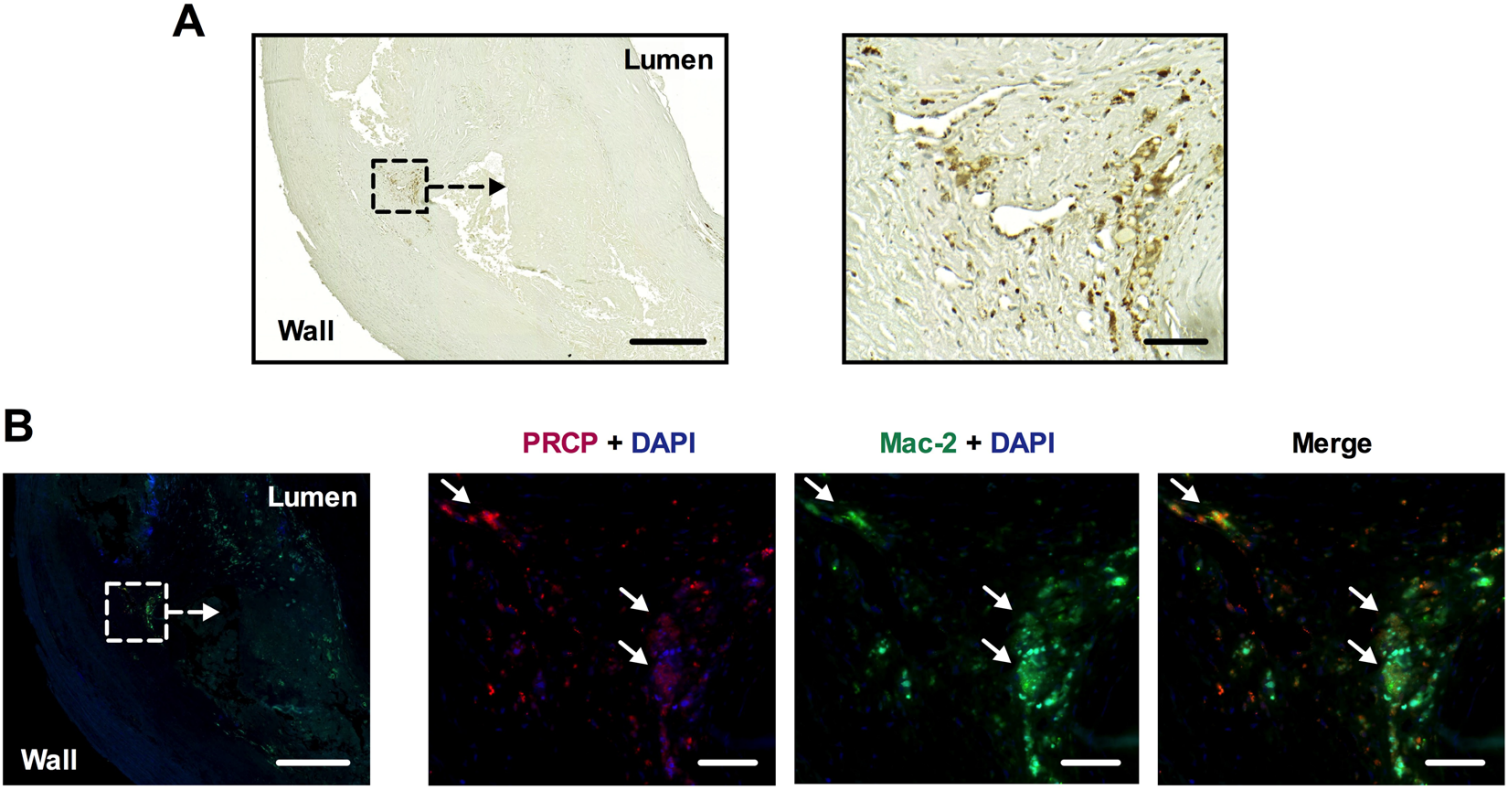
The expression and localization of PRCP in the human atherosclerotic plaque. (**A**) Immunohistochemical staining of PRCP (brown color) in a carotid endarterectomy sample. Scale bars, 500 µm (left) and 50 µm (right). (**B**) A consecutive section of the carotid sample was immunofluorescently stained for PRCP (red) and Mac-2 (green), and counterstained with DAPI. Cells that clearly express both PRCP and Mac-2 are indicated by white arrows. Scale bars, 500 µm (left) and 50 µm (right).

To gain further insights into the regulation of POMC processing in atherosclerosis, plaque samples were investigated for the expression of major enzymes involved in the proteolytic cleavage of POMC into biologically active peptides. The analyzed genes were PCSK1 and PCSK2 that encode PC1/3 and PC2, respectively, and regulate the first steps of POMC cleavage as well as CPE and PAM, which are more critical in the formation of mature α-MSH. Plaque samples were also screened for PRCP gene that was recently identified to inactivate α-MSH by degradation^5^. Firstly, POMC expression was found to negatively associate with CPE in carotid and abdominal plaque samples (Supplementary Fig. IIIA and B), while the correlation between POMC and PRCP was positive in carotid samples (Supplementary Fig. IIIA). Femoral plaques showed no distinct correlation between POMC and its processing enzymes (Supplementary Fig. IIIC). Secondly, gene expression levels of CPE, PAM and PRCP were studied between control samples and different plaque types as these genes are centrally involved in the maturation and degradation of α-MSH. CPE expression was specifically down-regulated (FC = −1.3, P = 0.03) in carotid plaques compared to LITA controls (Fig. 7A). PAM expression was significantly reduced in abdominal (FC = −1.4, P = 0.0001) and femoral (FC = −1.3, P = 0.002) plaques (Fig. 7B). In contrast, PRCP was consistently up-regulated (FC = 1.4–1.6) in all arterial beds (Fig. 7C). Further analysis of type V and VI advanced lesions revealed that CPE transcript levels were reduced (FC = −1.3, P = 0.04) and PRCP levels increased (FC = 1.3, P = 0.003) in unstable atherosclerotic plaques compared to stable samples (Fig. 7D,F), while PAM expression did not significantly differ between stable and unstable plaques (Fig. 7E). Gene association analyses showed that CPE expression correlated negatively with macrophage-specific signature, most notably with M2 macrophage markers, and positively with SMC-rich plaque signature (Supplementary Fig. IV). Likewise, PAM mRNA levels correlated negatively with M1/M2 macrophage markers and positively with SMC markers (Supplementary Fig. V), whereas completely opposite patterns were observed for PRCP (Supplementary Fig. VI). In general, these association patterns were strongest in carotid plaques (Supplementary Figs IV–VI). Lending support to the role of α-MSH in reverse cholesterol transport, CPE and PAM showed negative correlation with ABCA1, ABCG1 and SCARB1 in all plaque types. (Supplementary Fig. VII). PRCP transcript levels correlated positively with the reverse cholesterol transporters particularly in carotid plaque samples (Supplementary Fig. VII). Lastly, PRCP, which showed most significant changes at the mRNA level, was further studied by immunohistochemistry in the human atherosclerotic plaque. PRCP protein was expressed in the atherosclerotic plaque and it mainly localized to Mac-2 positive macrophages (Fig. 8).

## Discussion

The present study aimed at investigating the expression of POMC and POMC-related enzymes in human atherosclerosis. Here, we demonstrate that in POMC is not only expressed at the mRNA level but also processed into mature and biologically active cleavage product, namely α-MSH, in the atherosclerotic plaque. The expression of POMC-processing enzymes associated with plaque stability in a congruent manner, pointing towards a possible reduction of α-MSH in unstable advanced plaques. Lastly and consistent with our previous findings, POMC expression correlated positively with the reverse cholesterol transporters ABCA1, ABCG1 and SCARB1 in atherosclerotic plaques. However, in whole blood samples, the correlations were negative with signs of regulatory disturbance occurring after the development of CAD.

Our results indicate that POMC expression correlates with macrophage-rich gene signature in atherosclerotic plaque, suggesting that macrophages are a possible source of the pro-hormone POMC and its cleavage products. This notion is further supported by the finding of co-localization of α-MSH with the macrophage marker Mac-2 in immunofluorescence staining. Indeed, considering POMC production in different leukocyte subpopulations, the largest body of evidence demonstrates that macrophages are capable of producing α-MSH and that the rate of production is responsive to acute inflammatory stimuli^19–21^. The observations that femoral plaque samples showed higher POMC expression than atherosclerosis-free control arteries and that unstable plaques had similarly increased POMC transcript levels in comparison with stable plaques indicate that atherosclerosis induces changes in the regulation of POMC expression. Up-regulation of POMC had also resulted in blunted correlation patterns with macrophage and SMC markers in these samples, which points to a possible disturbance in POMC processing or to enhanced degradation of α-MSH. This notion is supported by the finding of discordant POMC and α-MSH expression levels in the aorta of Apoe^−/−^ mice, *i.e*. increased POMC and falling α-MSH level in response to diet-induced atherosclerosis. The mouse data provides persuasive evidence that POMC is up-regulated as a compensatory response to disturbed POMC processing and/or accelerated inactivation of α-MSH. Further research will be fundamental to explore how atherosclerosis affects POMC processing in mice and humans. It is well-known that local formation of α-MSH necessitates sequential actions of several enzymes in the biosynthetic pathway. PC1/3 and PC2 operate upstream in the pathway, while processing of ACTH to mature α-MSH is driven by CPE, PAM and NAT^3^. In view of the fact that POMC gives rise to a versatile array of bioactive peptides, it is clear that POMC gene has a complex promoter/enhancer structure and its expression is tightly regulated by numerous transcription factors^22^. Accordingly, the expression of CPE, PAM and NAT might more accurately reflect changes in the biosynthesis rate of α-MSH.

Another line of evidence from the present study strengthens the concept that α-MSH and its cognate receptor MC1R contribute to the regulation of reverse cholesterol transport. We had previously observed that MC1R expression correlates with ABCA1, ABCG1 and SCARB1 in human and mouse atherosclerotic plaques and that α-MSH has also a functional effect on reverse cholesterol transport by activating MC1R in macrophages^10^. The present findings indicate a positive correlation between POMC and the cholesterol transporter genes in carotid plaques. ABCA1 and ABCG1 initiate the first step of reverse cholesterol transport and counter-balance the cholesterol burden of macrophage-derived foam cells, thus providing protection against atherosclerosis and development of vulnerable plaque phenotype^11–13^. SCARB1 is considered as the HDL receptor due to its central role in regulating cholesterol uptake from HDL particles in the liver^23,24^. It also mediates bidirectional lipid transport in macrophages and is associated with the development of atherosclerosis^25,26^. Against this background, the expression of POMC in lesional macrophages might provide an atheroprotective mechanism by regulating reverse cholesterol transport in an autocrine or paracrine manner in the plaque environment.

Interestingly, the expression of POMC in whole blood samples correlated with reverse cholesterol transporters and the presence of CAD seemed to abrogate this association. However, the direction of correlation was negative and thereby contradicts the findings in arterial plaque samples. One possible explanation for this discrepancy might be that the regulation between POMC and reverse cholesterol transporters is completely different in circulating leukocytes compared to lesional macrophages. Another finding from the association analyses with non-CAD and CAD samples was that the correlation appeared in whole blood samples and not in circulating monocyte fractions. POMC transcripts have been found in a variety of immune cells, including monocytes, neutrophils and lymphocytes^27–29^. Thus, it is conceivable that the interaction between POMC and ABCA1/ABCG1 may occur primarily in neutrophils or in lymphocytes. However, little is known about the role and regulation of reverse cholesterol transporters in other leukocyte subsets than monocytes and their descendant macrophages^11,25^. The lack of association between POMC and ABCA1/ABCG1 in monocytes could be on the other hand caused by sample processing for monocyte isolation and its influence on gene expression. Clearly, further studies are warranted to investigate how POMC and its processing products regulate ABCA1/ABCG1 expression in circulating leukocytes and what is the pathological significance of the observed lack of association in CAD.

In addition to subtle changes in POMC expression between different plaque types, further analyses uncovered that the enzymes responsible for the processing and maturation of α-MSH are down-regulated in atherosclerotic plaques, while α-MSH inactivating PRCP expression is increased in plaque samples. Similar changes associated also with unstable plaque phenotype, suggesting that POMC processing might be further disturbed during plaque progression to a vulnerable lesion at high risk for rupture. In terms of correlation with pro-inflammatory M1 type and anti-inflammatory M2 type macrophage markers, no clear-cut polarization towards either macrophage type was observed for the studied enzymes except for CPE, which mostly correlated with M2 type markers. Thus, CPE-driven α-MSH maturation could occur primarily in anti-inflammatory M2 type macrophages, which are typically enriched in regressing lesions and are likely to be atheroprotective^30,31^. Reduced CPE and PAM expression and increased PRCP expression are likely to reduce the local availability of α-MSH in the lesions. The finding of reduced α-MSH expression in advanced lesions of Apoe^−/−^ mice lends support to the view that its amount might decline during the progression of human atherosclerosis. Given that pharmacological or genetic α-MSH overexpression promotes an anti-inflammatory plaque phenotype and limits atherosclerosis^10,14,15^, reduced α-MSH level could induce a reverse phenotype and thus be a contributing factor for the development of vulnerable plaque. However, the expressional changes of CPE, PAM and PRCP are indirect markers and provide only suggestive evidence that α-MSH bioavailability might be affected. CPE and PAM not only process ACTH into biologically active α-MSH but they are also involved in the generation of other POMC-derived products including β-MSH, γ-MSH and β-endorphins^3^. Secondly, PRCP was only recently identified to inactivate α-MSH^5,32^, while the earlier evidence demonstrate its central role in the metabolism of angiotensin II and III as well as in the activation of prekallikrein to kallikrein^33,34^. Even if other peptide systems are also modulated, the observed changes in PAM, CPE and PRCP transcript levels suggest a synergistic effect on lowering tissue α-MSH levels.

From a drug development perspective, PRCP represents an attractive therapeutic target to augment the bioavailability of α-MSH for the treatment of cardiometabolic diseases^35,36^. For example, PRCP deficient mice exhibit reduced food intake and resistance to diet-induced obesity due to elevated α-MSH levels^5^. Similar effects have been also reproduced by using small-molecule inhibitors of PRCP^5,37^. Extending the experimental findings to a clinical perspective, plasma concentration of PRCP was found to associate with obesity, diabetes and signs of atherosclerotic plaque formation^38^. These findings are in agreement with the present study showing up-regulated PRCP expression particularly in unstable advanced plaques. However, owing to its role in processing vasoactive peptides, PRCP has been also implicated in the regulation of blood pressure, angiogenesis and endothelial inflammation^39–41^, highlighting the complexity of the involved molecular pathways. Further research will be therefore fundamental to characterize the overall outcome and safety of PRCP inhibition.

As a potential limitation of the study, plaque samples were compared to atherosclerosis-free LITAs instead of corresponding normal arteries from carotid, aortic and femoral regions due to ethical issues. Because of the systemic nature of atherosclerosis, LITA samples and their gene expression profile may be affected by pro-inflammatory signaling and other humoral mechanisms that are driving atherosclerosis. Nevertheless, the most significant findings of the present study were derived from the comparisons between stable and unstable advanced lesions, which are not confounded by the same factor. A considerable limitation is, however, that most of the findings are based on mRNA level analyses and it remains to be determined whether the observed changes are also present at the protein level in human atherosclerotic plaques.

In conclusion, the present study demonstrates that the expression of POMC and the enzymes involved in the processing of its cleavage product, α-MSH, associate with plaque stability. The results also further consolidate the role of POMC-derived α-MSH in regulating reverse cholesterol transport and reveal a possible disturbance in this regulatory pathway after manifestation of CAD. Hence, pharmacological means to increase α-MSH bioavailability, *e.g. via* inhibition of PRCP, could provide a therapeutic strategy to treat atherosclerotic cardiovascular disease, but future mechanistic experiments will be instrumental to clarify the role of PRCP in atherosclerosis.

## Materials and Methods

### Tampere vascular study (TVS) samples

Endarterectomy samples were obtained from femoral and carotid arteries, and abdominal aortas of patients fulfilling the following inclusion criteria: (1) carotid endarterectomy because of asymptomatic or symptomatic and hemodynamically significant (>70%) carotid stenosis or (2) femoral or (3) aortic endarterectomy with aortoiliac or aortobifemoral bypass because of symptomatic peripheral arterial disease^18,42^. The left internal thoracic artery (LITA) samples, obtained during coronary artery bypass surgery, served as controls. Gene expression was analyzed from carotid (n = 29), abdominal aortic (n = 15), and femoral (n = 24) plaques and histologically atherosclerosis-free LITAs (n = 24). The type V and VI atherosclerotic lesions, according to the American Heart Association recommendation^43^, were further histologically classified as stable or unstable according to the presence of fissure, rupture, hemorrhage and thrombosis. The study was approved by the Ethics Committee of Tampere Hospital District and conducted according to the declaration of Helsinki, and the study subjects gave informed consent.

### Whole blood and monocyte samples

TVS whole blood and monocyte fractions were collected and used for genome-wide analysis of gene expression during 2008. The samples were selected from a larger population-based cross-sectional study comprising patients subjected to an exercise test at Tampere University Hospital and thereafter treated according to the Finnish Current Care Guidelines^44^. RNA was isolated from the whole blood and monocyte fractions of individuals with angiographically verified coronary artery disease (CAD) (n = 55) and without coronary artery lesions (non-CAD) (n = 45). Monocytes were isolated from the whole blood samples by Ficoll-Paque density-gradient centrifugation (Amersham Pharmacia Biotech UK Limited, Buckinghamshire, England).

### Mouse atherosclerosis samples

Mouse atherosclerosis samples were derived from 6-month-old female apolipoprotein E deficient (Apoe^−/−^) mice. Mice were fed ad libitum a regular chow diet or a cholesterol-rich diet (RD Western Diet, D12079B, Research Diets Inc, NJ, USA) for 12 weeks to promote atherosclerosis. At the end of the experiment, mice were euthanized *via* CO_2_ asphyxiation and aortic roots were harvested and fixed overnight in formalin. The animal experiment was approved by the local ethics committee (Animal Experiment Board in Finland, License Number: ESAVI/6280/04.10.07/2016) and conducted in accordance with the institutional and national guidelines for the care and use of laboratory animals.

### RNA isolation and genome-wide expression analysis

The fresh arterial tissue samples were soaked in RNALater solution (Ambion Inc., Austin, TX, USA) and isolated with Trizol reagent (Invitrogen, Carlsbad, CA, USA) and the RNAEasy Kit (Qiagen, Valencia, CA, USA). RNA from whole blood fractions was extracted with PAXgene tubes (BD, Franklin Lakes, NJ) and PAXgene Blood RNA Kit (Qiagen) with DNase Set. Peripheral mononuclear cells were isolated from the whole blood samples by Ficoll-Paque density-gradient centrifugation (Amersham Pharmacia Biotech UK Limited, Buckinghamshire, England) and total RNA was then extracted using RNeasy Mini Kit (Qiagen). Thereafter, 200 ng aliquots of total RNA from each sample were converted to cDNA (Illumina RNA Amplification kit, iAmbion, Inc., Austin, TX, USA). The expression levels of arterial and whole blood samples were analyzed with Illumina HumanHT-12 v3 Expression BeadChip (Illumina, San Diego, CA). Monocyte samples were analyzed using Sentrix Human-6 Expression BeadChips (Illumina). After background subtraction, raw expression data were imported into R version 3.1.1 (http://www.r-project.org/), log2 transformed and normalized by the locally estimated scatterplot smoothing normalization method implemented in the R/Bioconductor package Lumi (www.bioconductor.org). The accuracy of this method has been previously tested in our TVS validation study, showing good correlation between expression measurements by microarray and RT-PCR methods (r = 0.87, y = 0.151 + 0.586×)^16^.

### Immunohistochemistry and immunofluorescence

Formalin-fixed and paraffin-embedded human endarterectomy and mouse aortic root sections were immunostained using a rabbit polyclonal antibody against α-MSH (dilution 1:2000, Immunostar, Inc., Hudson, WI, USA, Cat No: 20074)^9^ or a rabbit polyclonal antibody against PRCP (dilution 1:250, Sigma-Aldrich, St. Louis, MO, Cat No: HPA017065). Briefly, sections were deparaffinized, rehydrated, and exposed to antigen-retrieval protocol with 10 mM sodium citrate buffer (pH 6). Sections were thereafter quenched for endogenous peroxidase activity using 1% H_2_O_2_ in Tris-buffered saline (TBS), blocked with 10% normal horse serum and incubated with the primary antibody (dilution 1:2000) overnight at 4 °C. Control sections were incubated with a rabbit IgG isotype control antibody (Novus Biologicals, Littleton, CO, USA, Cat No: NBP2-24891) (Supplementary Fig. VIII and IX). Primary antibody binding was detected with a horseradish peroxidase-conjugated secondary antibody and diaminobenzidine (ABC kit, Vector Labs, Burlingame, USA). For double immunofluorescence, sections were incubated additionally with a rat Mac-2 antibody (Cedarlane Labs, Burlington, ON, Canada) and then with fluorochrome-conjugated secondary antibodies (anti-rabbit Alexa Fluor 647 and anti-rat Alexa Fluor 488, Jackson ImmunoResearch, West Grove, USA). Sections were counterstained with hemotoxylin (CarlRoth, Karsruhe, Germany) or DAPI (Fluoroshield mounting medium, Abcam, Cambridge, UK), coverslipped and then scanned with a digital slide scanner (Pannoramic 250 or Pannoramic Midi with Zeiss filter sets for FITC, Cy5 and DAPI, 3DHISTECH Kft., Budapest, Hungary).

### Quantitative RT-PCR

Total RNA from the aorta of Apoe^−/−^ mice was extracted (Qiagen, Venlo, Netherlands) and reverse-transcribed (Takara Clontech). Quantitative RT-PCR was performed using SYBR Green protocols (Kapa Biosystems, MA, USA) on an Applied Biosystems 7300 Real-Time PCR system. Samples were run in duplicate. Target gene mRNA expression levels were normalized to the geometric mean of ribosomal protein S29 and β-actin using the comparative ΔCt method and are presented as relative transcript levels (2^−ΔΔCt^). Primer sequences were as follows: POMC (forward): 5′-caagccggtgggcaagaaacg-3′ and POMC (reverse): 5′-ctaatggccgctcgccttccag-3′. The primers for S29 and β-actin have been previously reported^45^.

### Statistical analysis

Statistical analyses were performed with GraphPad Prism 6 (GraphPad Software Inc., La Jolla, CA, USA). Comparisons of gene expression between controls and cases were performed using log-transformed data and Student’s t test, the nonparametric Mann-Whitney U test or one-way ANOVA and Bonferroni *post hoc* test. Pearson or nonparametric Spearman correlation coefficients were calculated for gene associations based on D’Agostino-Pearson ominibus normality test results. All data are presented as mean ± standard error of the mean (SEM). A two-tailed P value of <0.05 was considered statistically significant.

## Electronic supplementary material

Supplementary information

## Data Availability

All data analyzed during this study are included in the published article and its Supplementary Information file or can be requested from the corresponding author.
