# Supplementary material for: Pro-opiomelanocortin and its Processing Enzymes Associate with Plaque Stability in Human Atherosclerosis – Tampere Vascular Study
Source: Sci Rep. 2018 Oct 10;8:15078. doi: 10.1038/s41598-018-33523-7 (PMC6180013; doi:10.1038/s41598-018-33523-7)
Supplement: Supplementary file 1 — Supplementary information [file 41598_2018_33523_MOESM1_ESM.pdf]

SUPPLEMENTARY INFORMATION

Pro-opiomelanocortin and Its Processing Enzymes Associate with Plaque Stability in Human Atherosclerosis – Tampere Vascular Study

Petteri Rinne, Leo-Pekka Lyytikäinen, Emma Raitoharju, James J. Kadiri, Ivana Kholova, Mika Kähönen, Terho Lehtimäki *, Niku Oksala *

* Equal contribution

Corresponding Author: Niku Oksala, MD, PhD, DSc
Fimlab Laboratories, Department of Surgery and Clinical Chemistry, Tampere University Hospital, Finn-Medi 2, 3rd Floor, PO Box 2000, FI-33521 Tampere, Finland. E-mail: nikuoksala@gmail.com.

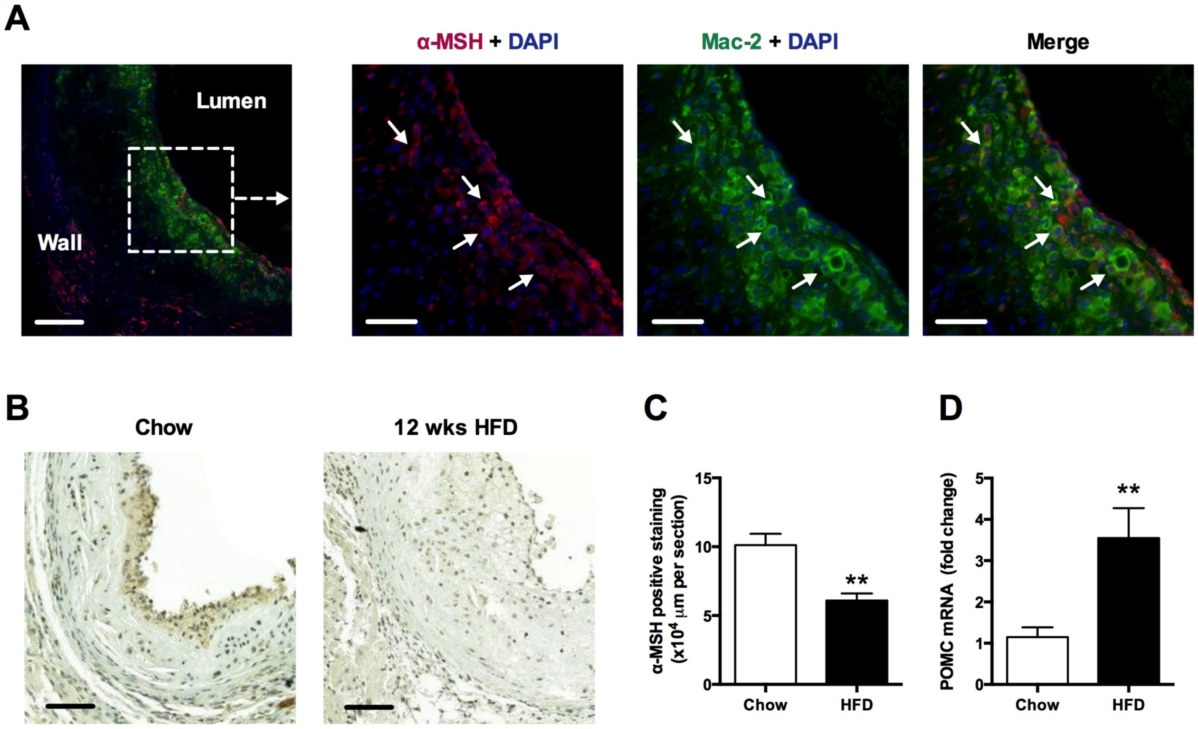

Supplementary Figure I. α-MSH expression localizes in lesional macrophages and is reduced in advanced atherosclerotic plaques of Apoe$^{-/-}$ mice. (**A**) Representative double immunofluorescence staining of α-MSH and Mac-2 in the aortic root section of chow-fed Apoe$^{-/-}$ mouse. The section was counterstained with DAPI. Some of the cells that express both α-MSH and Mac-2 are indicated by white arrows. Scale bars, 200 μm (left) and 50 μm (right). (**B**) Representative α-MSH-immunostained (brown color) aortic root sections from Apoe$^{-/-}$ mice fed a regular chow diet or atherogenic high-fat diet (HFD) for 12 weeks. Scale bars, 100 μm. (**C**) Quantification of absolute (in μm^2 per section) α-MSH-positive are in aortic root sections of chow- and HFD-fed Apoe$^{-/-}$ mice. (**D**) Quantitative RT-PCR analysis of POMC expression (expressed as fold change compared to chow-fed mice) in the aorta of chow- and HFD-fed Apoe$^{-/-}$ mice. * $P<0.05$ and ** $P<0.01$ versus chow-fed mice (Mann-Whitney U test). Data are mean ± SEM, n=6-9 mice per group.

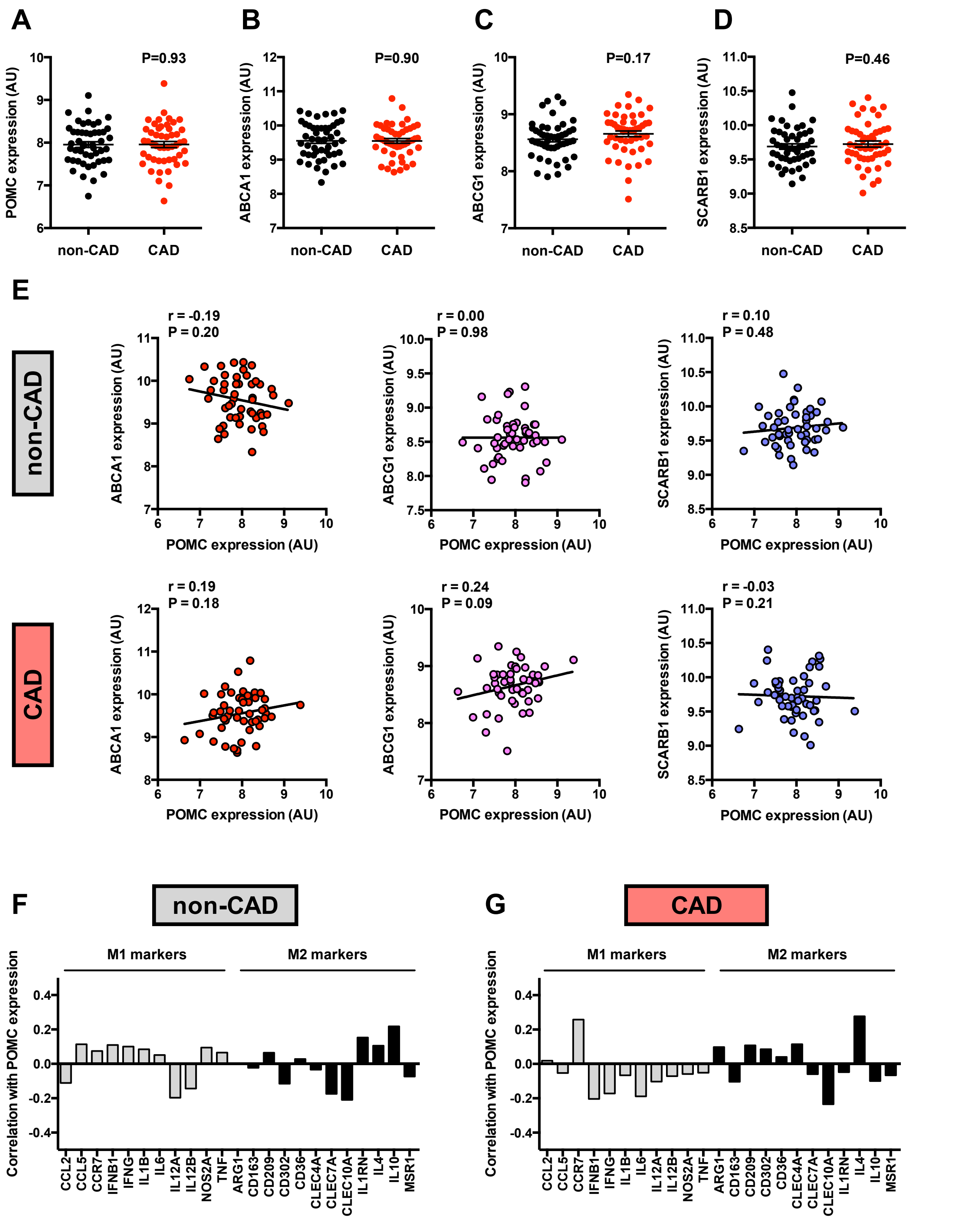

Supplementary Figure II. POMC expression and its association with reverse cholesterol transporters in monocyte samples. (**A-D**) POMC, ABCA1, ABCG1 and SCARB1 mRNA levels in monocyte samples of patients with history of coronary artery disease (CAD) and of individuals without coronary lesions (non-CAD). Exact P values are presented in the graphs. (**E**) Correlations between POMC expression and ABCA1, ABCG1 and SCARB1 expression in whole blood samples from non-CAD and CAD individuals. (**F** and **G**) Correlation between POMC mRNA levels and established M1 (grey columns) and M2 (black columns) macrophage markers in non-CAD (n=48) and CAD (n=47) monocyte samples. Pearson correlation coefficient (r) and P values are presented in the graphs.

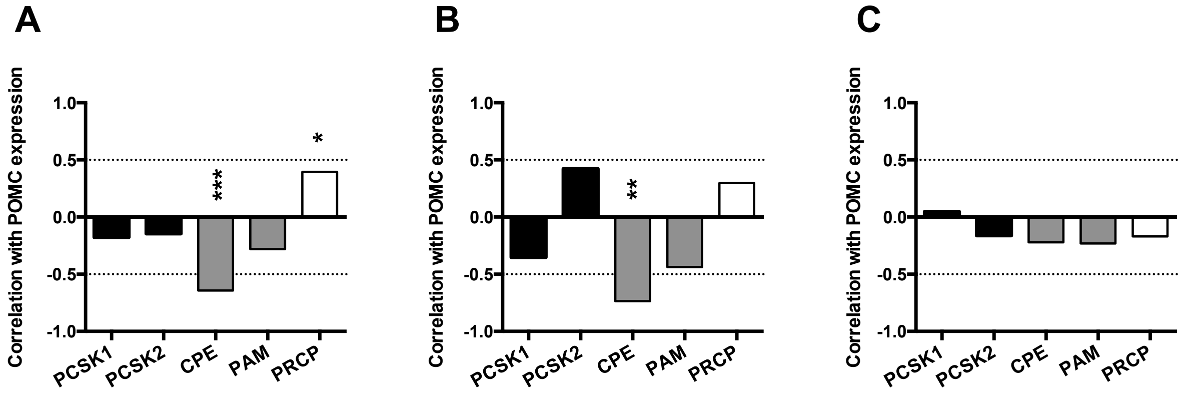

Supplementary Figure III. Correlation between POMC and POMC-processing enzyme mRNA levels in human atherosclerotic plaques. Correlation of POMC expression with the enzymes involved in the processing of POMC and degradation of α-MSH in carotid (**A**), abdominal (**B**) and femoral (**C**) atherosclerosis samples. PCSK1/2, proprotein convertase subtilisin/kexin type 1/2; CPE, carboxypeptidase E; PAM, peptidylglycine α-amidating monooxygenase; PRCP, prolylcarboxypeptidase. Pearson correlation coefficient (r) values are presented in the column graphs. * $P<0.05$, ** $P<0.01$ and *** $P<0.001$ for correlation significances. n=29 (carotid), n=15 (abdominal, n=24 (femoral).

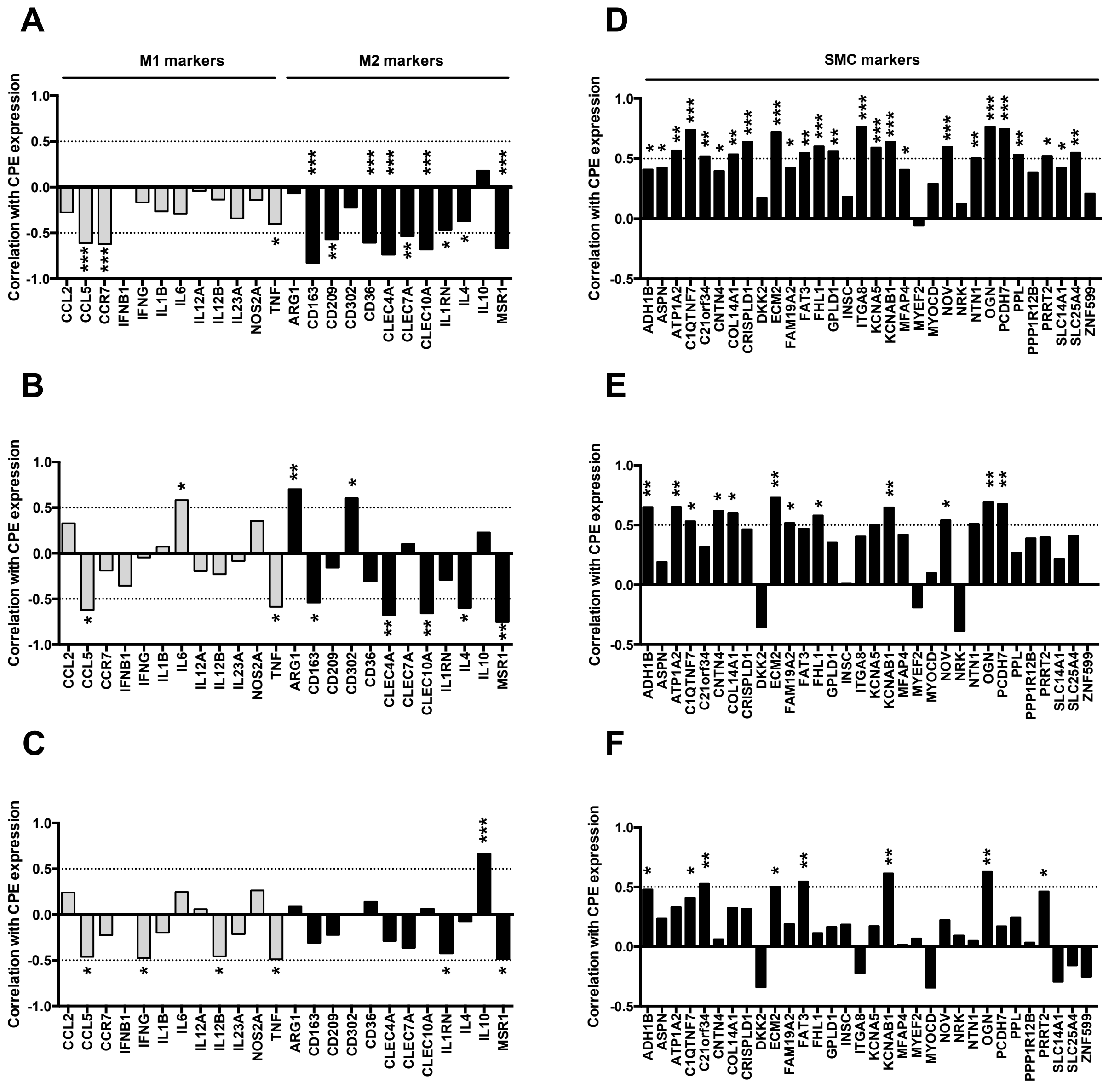

Supplementary Figure IV. Correlation between carboxypeptidase E (CPE) mRNA levels and M1/M2 macrophage markers and smooth muscle cell (SMC) markers. (**A-C**) (**A-C**) Correlation between CPE mRNA levels and established M1 (grey columns) and M2 (black columns) macrophage markers in carotid (**A**), abdominal (**B**) and femoral (**C**) plaque samples. (**D-F**) Correlation between CPE mRNA levels and smooth muscle cell markers in carotid (**A**), abdominal (**B**) and femoral (**C**) plaque samples. Pearson correlation coefficient (r) values are presented in the column graphs. * P<0.05, ** P<0.01 and *** P<0.001 for correlation significances. n=29 (carotid), n=15 (abdominal), n=24 (femoral).

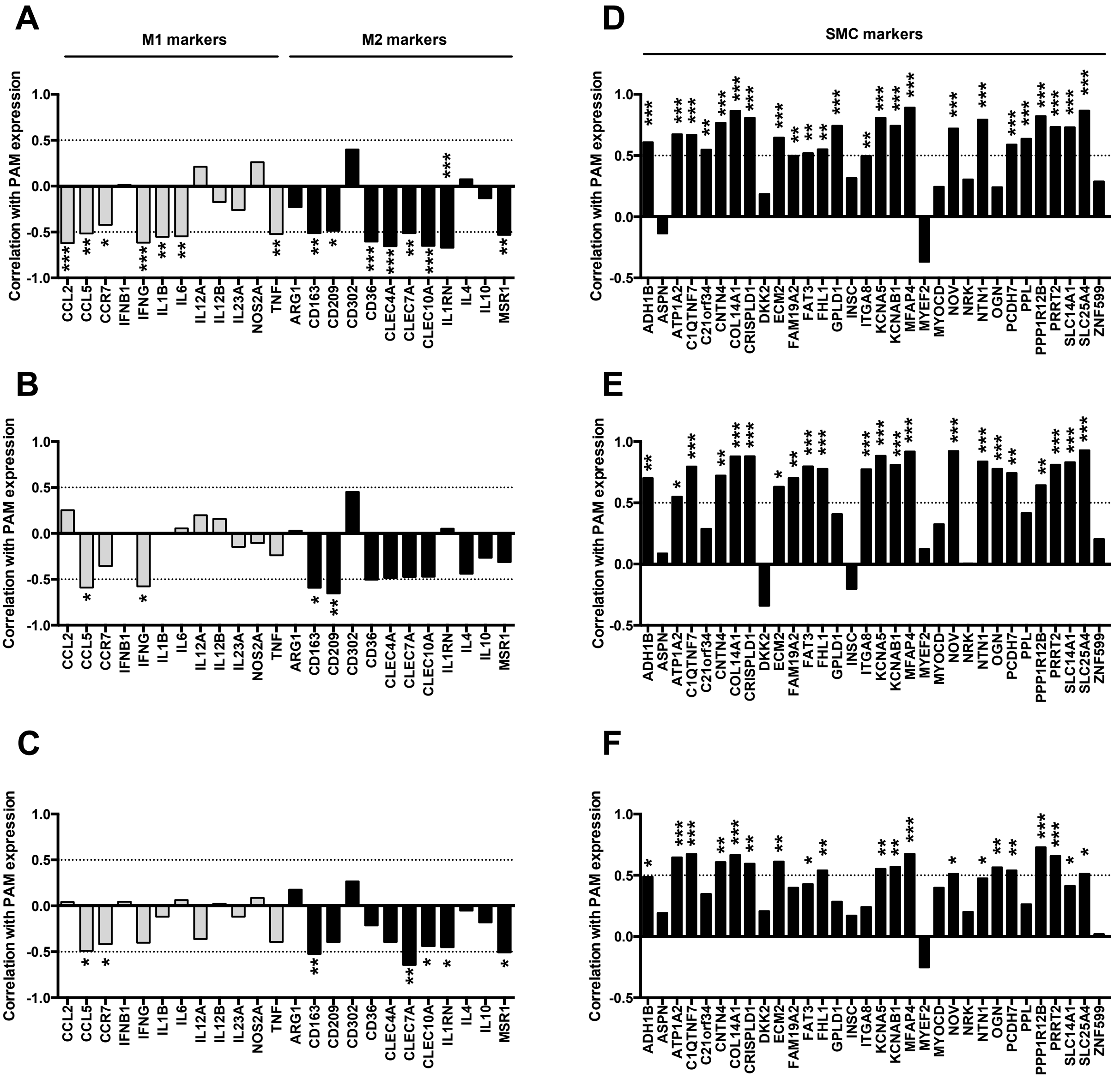

Supplementary Figure V. Correlation between peptidylglycine α-amidating monooxygenase (PAM) mRNA levels and M1/M2 macrophage markers and smooth muscle cell (SMC) markers. (**A-C**) Correlation between PAM mRNA levels and established M1 (grey columns) and M2 (black columns) macrophage markers in carotid (**A**), abdominal (**B**) and femoral (**C**) plaque samples. (**D-F**) Correlation between PAM mRNA levels and smooth muscle cell markers in carotid (**A**), abdominal (**B**) and femoral (**C**) plaque samples. Pearson correlation coefficient (r) values are presented in the column graphs. * P<0.05, ** P<0.01 and *** P<0.001 for correlation significances. n=29 (carotid), n=15 (abdominal), n=24 (femoral).

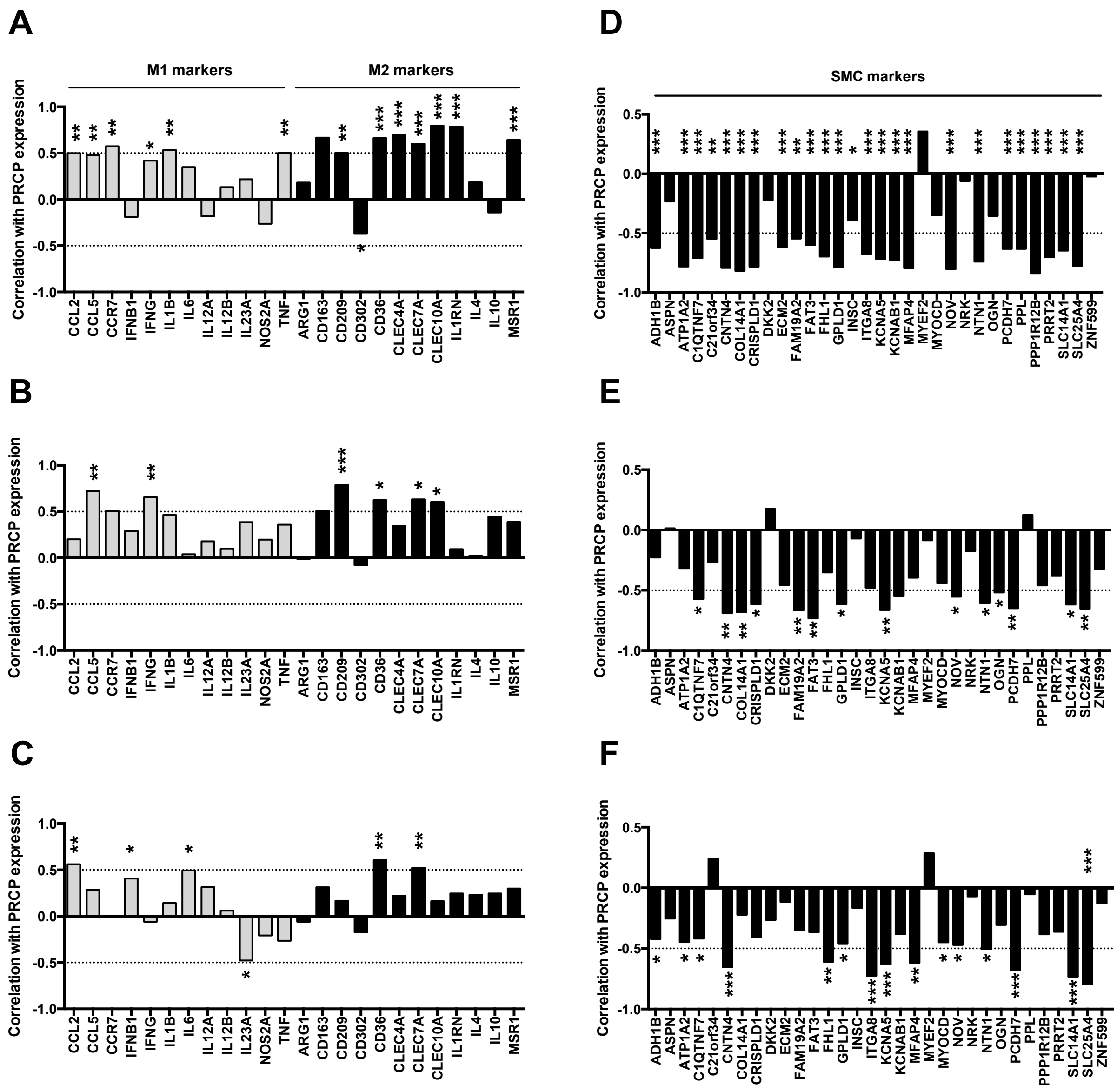

Supplementary Figure VI. Correlation between prolylcarboxypeptidase (PRCP) mRNA levels and M1/M2 macrophage markers and smooth muscle cell (SMC) markers. (**A-C**) Correlation between PRCP mRNA levels and established M1 (grey columns) and M2 (black columns) macrophage markers in carotid (**A**), abdominal (**B**) and femoral (**C**) plaque samples. (**D-F**) Correlation between PRCP mRNA levels and smooth muscle cell markers in carotid (**A**), abdominal (**B**) and femoral (**C**) plaque samples. Pearson correlation coefficient (r) values are presented in the column graphs. * P<0.05, ** P<0.01 and *** P<0.001 for correlation significances. n=29 (carotid), n=15 (abdominal), n=24 (femoral).

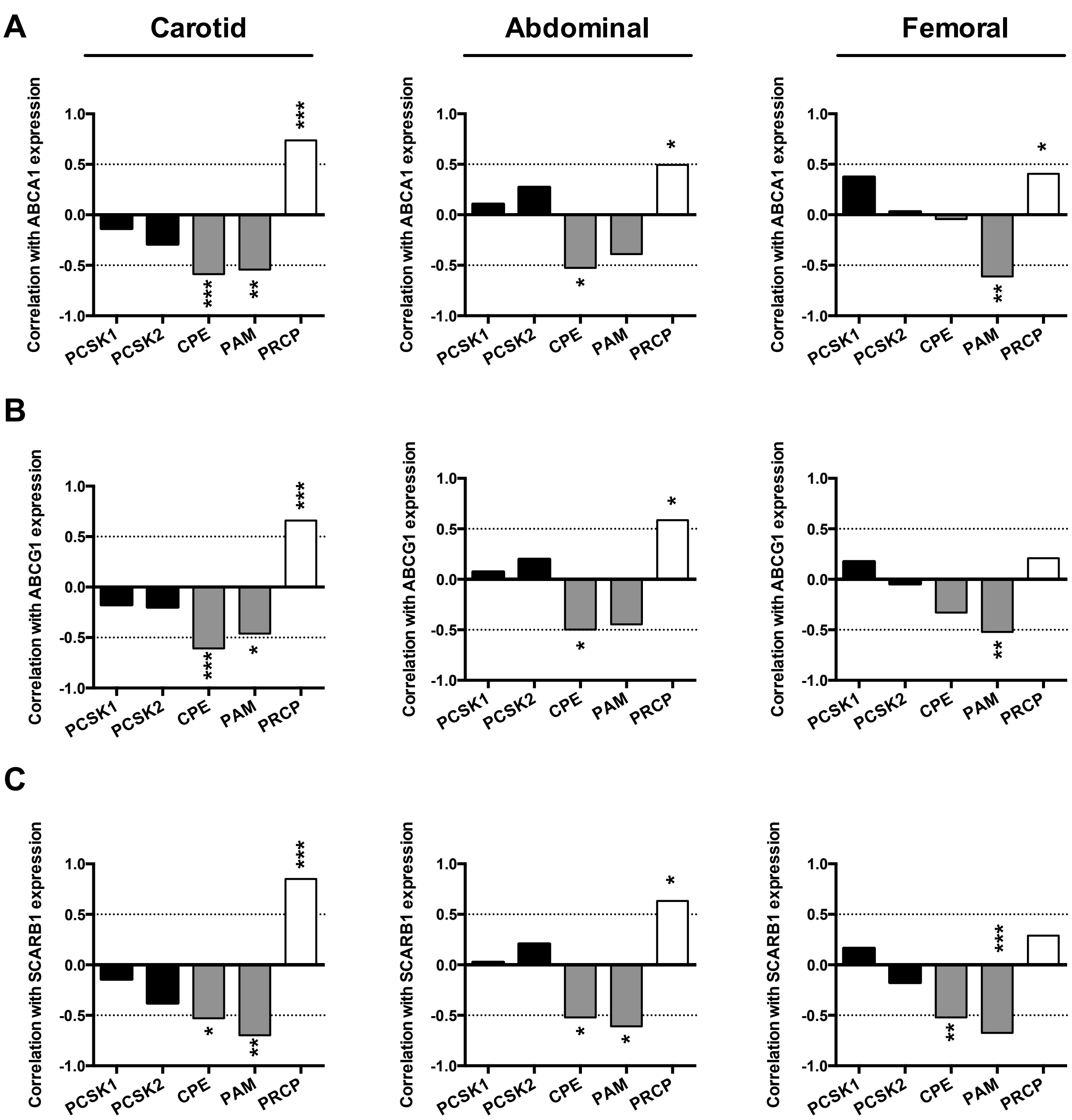

Supplementary Figure VII. Correlation between reverse cholesterol transporter (ABCA1, ABCG1 and SCARB1) and POMC-processing enzyme mRNA levels in human atherosclerotic plaques. (**A**) Correlation of ABCA1 expression with the POMC-processing enzyme mRNA levels in carotid (**left**), abdominal (**middle**) and femoral (**right**) plaque samples. (**B**) Correlation of ABCG1 expression with the POMC-processing enzyme mRNA levels. (**C**) Correlation of SCRAB1 expression with the POMC-processing enzyme mRNA levels. PCSK1/2, proprotein convertase subtilisin/kexin type 1/2; CPE, carboxypeptidase E; PAM, peptidylglycine α-amidating monooxygenase; PRCP, prolylcarboxypeptidase. Pearson correlation coefficient (r) values are presented in the column graphs. * $P<0.05$, ** $P<0.01$ and *** $P<0.001$ for correlation significances. n=29 (carotid), n=15 (abdominal), n=24 (femoral).

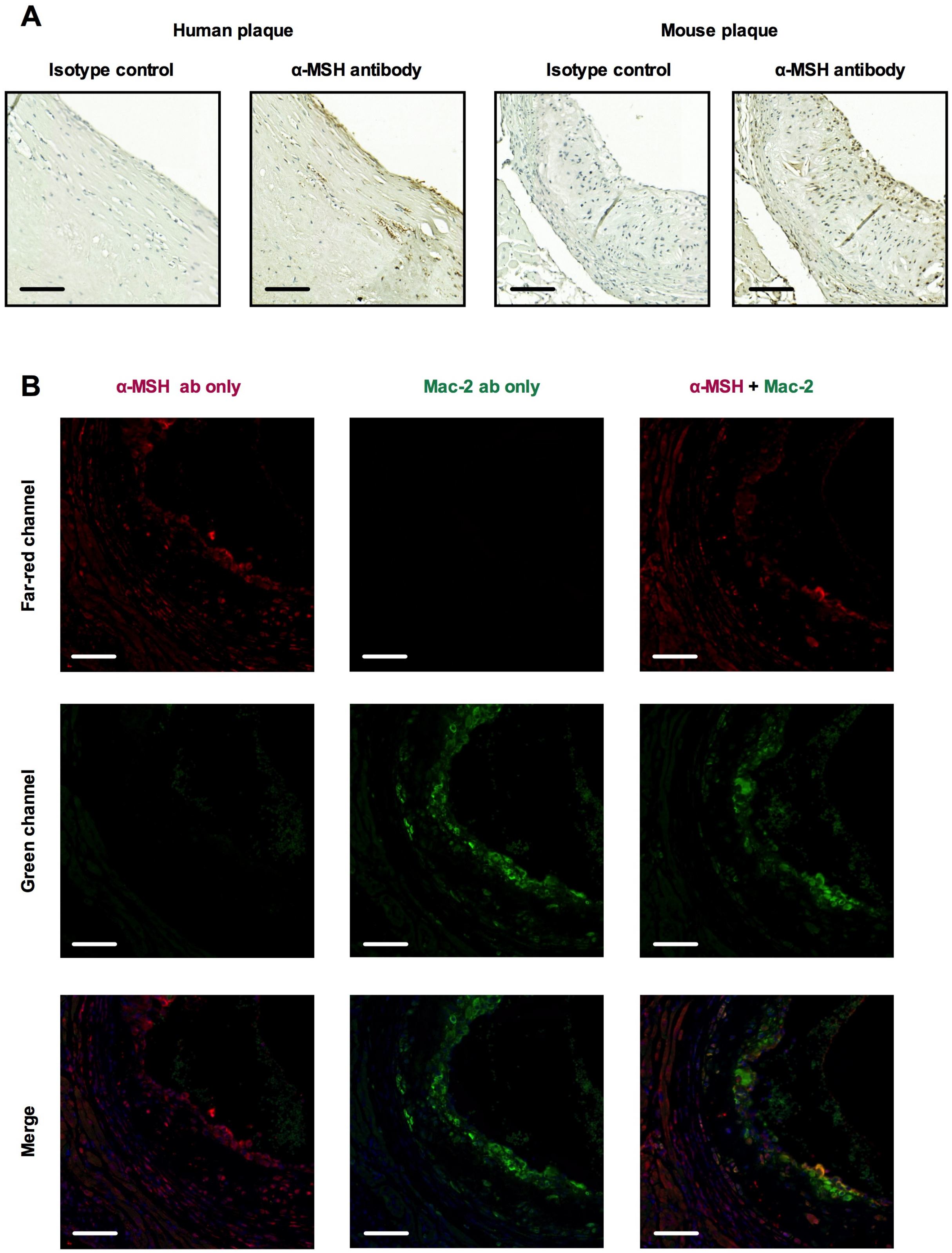

Supplementary Figure VIII. Controls for α-MSH immunohistochemistry and immunofluorescence. (**A**) Isotype controls for α-MSH were obtained by staining consecutive sections of human and mouse atherosclerotic plaques with α-MSH antibody or rabbit polyclonal isotype control antibody. (**C**) Appropriate single stain controls for α-MSH+Mac2 immunofluorescence staining in mouse atherosclerotic plaque. Scale bar, 100 µm in each panel.

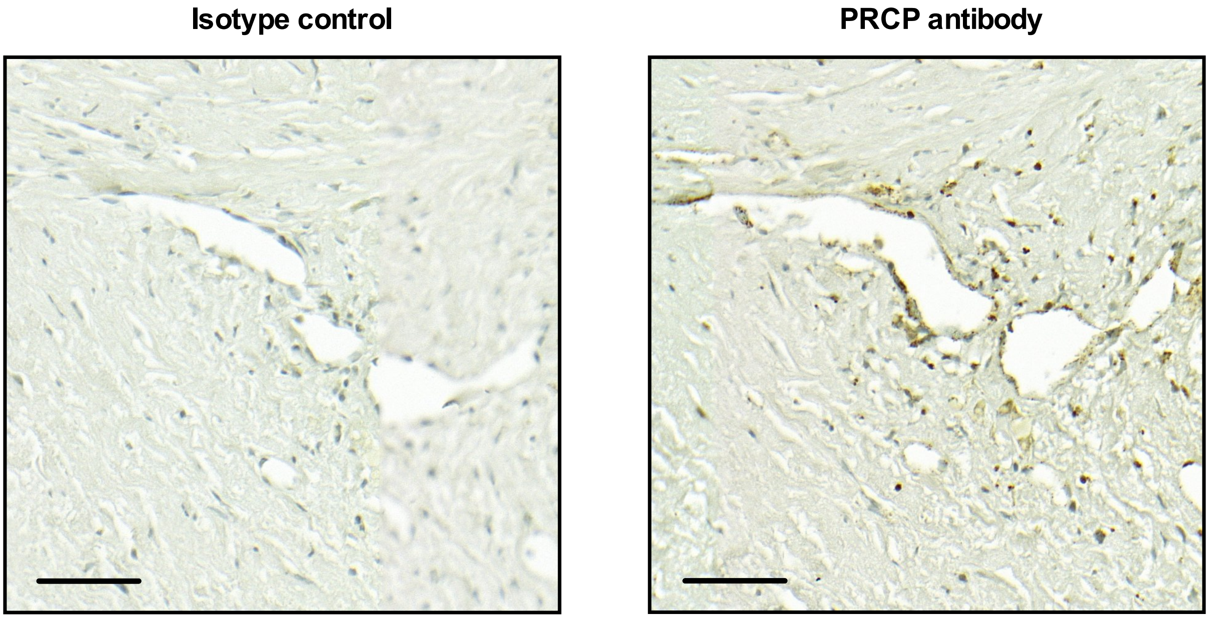

Supplementary Figure IX. Control staining for PRCP immunohistochemistry. Isotype control for PRCP was obtained by staining consecutive sections of a human carotid endarterectomy sample with PRCP antibody or rabbit polyclonal isotype control antibody. Scale bars, 50 µm.
